# Mental health support in German schools: a cross-sectional survey study of principals on demand vs. provision of support services

**DOI:** 10.3389/fpubh.2026.1760608

**Published:** 2026-06-24

**Authors:** Hannah Elisabeth Köhler, Judith Bauch, Kristin Rodney-Wolf, Franziska Greiner-Döchert, Katarina Heitz, Henrik Saalbach, Eva Baumann, Nina Tucholski, Julian Schmitz

**Affiliations:** 1 Department of Clinical Child and Adolescent Psychology, Faculty of Life Sciences, Leipzig University, Leipzig, Germany; 2 Department of Educational Psychology, Faculty of Education, Leipzig University, Leipzig, Germany; 3 Department of Journalism and Communication Research, Hanover University of Music, Drama and Media, Hanover, Germany

**Keywords:** Germany, guidance counseling, mental health support, school, school psychology, school social work, support structures

## Abstract

**Introduction:**

Student mental health concerns are rising globally and educational institutions constitute critical settings for the prevention and early intervention of these issues in youth. Yet, mental health support structures often vary between countries, regions, and schools and a systematic monitoring of provision and use of services is missing.

**Methods:**

To fill this gap, we surveyed 486 principals across 13 German federal states in spring 2024 with a questionnaire developed through literature review, expert interviews and pilot testing by an interdisciplinary expert committee. The instrument assessed the availability and demand (as estimated by principals) for guidance counseling, school social work, and school psychology services during the previous and current school years, and examined variations by school type (primary school, lower track secondary and comprehensive school, higher track secondary school, and special-needs school). The data was analyzed using *t*-tests, chi-square-tests and analysis of variance (ANOVA).

**Results:**

Findings revealed that demand increased compared to the previous school year, whereas provision remained unchanged. Although the majority of schools had access to school social workers and school psychology services, fewer than half employed guidance counselors. Where services were absent, demand was consistently high. Across all three domains, service provision failed to meet demand, which was up to twice as high as that offered by the services available. While resources varied across school types, no type consistently received more resources, though special-needs schools reported the highest level of both service provision and demand.

**Discussion:**

These findings reveal a significant mismatch between students' mental health needs and institutional capacity in German schools, underscoring the necessity to expand school-based services and to implement systematic, comparative monitoring at the international level for the optimal governance of health-care infrastructures.

## Introduction

Mental health, defined by the World Health Organization (WHO) as “a state of mental wellbeing that enables people to cope with stresses of life, realize their abilities, learn well and work well, and contribute to their community”, is an “integral component of health” (1). The foundation of good mental health is laid early in life, making child and adolescent wellbeing critical, while early-onset mental illness increases the risk of chronic and secondary disorders, social isolation and reduced life expectancy (2–5). Moreover, poor mental health in childhood is also linked to negative long-term socioeconomic outcomes, such as reduced academic performance and increased risk of future unemployment (6–8). Furthermore, untreated mental health issues among children and adolescents generate substantial long-term societal costs, rendering early intervention both socially responsible and economically imperative (9, 10). Despite these known high individual and societal costs, a recent meta-analysis indicates that 15.5% of European children and adolescents suffer from a mental disorder (11). The COVID-19 pandemic has further heightened the prevalence of mental health disorders among children and adolescents, underscoring an urgent need for expanded mental-health services (12, 13). Increasing prevalence rates have led the *Lancet Psychiatry Commission* to characterize the state of youth mental health as a global crisis, stressing the need for early intervention to prevent long term adverse outcomes for both individuals and societies (14).

### Mental health provision in schools

As one of the central social institutions in the lives of young people, schools constitute a pivotal context for community-based mental health services, as global research demonstrates (15–17). Owing to schools' daily interactions with students and the trust frequently placed in school staff by both students and families, they are uniquely positioned to identify early signs of psychological difficulties and to mitigate the adverse consequences of poor mental health among students (15). Mental health concerns are more likely to be recognized in school settings than in the community and screening meets fewer logistical barriers in schools (15, 16, 18). Integrating mental health prevention and interventions directly into the school context enhances access and adherence to the treatment and reduces stigma (15).

A systematic review of reviews showed that school-based interventions yield significant, positive effects that translate into practical benefits; e.g. improved academic performance through the development of social and emotional skills (17, 19). In addition, the implementation of psychoeducational content and programs within schools has proven beneficial, as they contribute to enhancing mental health literacy (the “knowledge and beliefs about mental disorders which aid their recognition, management or prevention” (20)) and reducing stigma among both students and staff (21). By fostering greater awareness and understanding of mental health issues, such initiatives facilitate the early recognition of psychological disorders and help to diminish barriers to appropriate treatment (21, 22).

Finally, teachers and school-based mental health staff play a vital role in referring students to specialist mental health care outside of school for conditions beyond school capacities, e.g. to outpatient psychotherapists or inpatient treatment (23, 24).

### National differences

While schools serve as an important setting for the promotion of mental health and the provision of mental health services, the scope, structure, and accessibility of such services vary considerably across countries and regions, reflecting differences in educational systems, health policies, and resource allocation. For instance, mental health support within the educational setting is robust in the Netherlands and Finland, but notably weaker in in other countries i.e. France (25–27). These disparities may interact with psychosocial services offered outside schools, as some countries (e.g., the UK, Finland) have extensive mental health services for youth in general, while others, such as the Netherlands, offer numerous inpatient treatment placements (28).

### The German school system

The subject of this study, Germany, is generally regarded as a well-resourced country in the domain of clinical child and adolescent mental health care, particularly due to its exceptionally high inpatient treatment capacity (28, 29). An additional setting in which mental health support is provided is German schools.

The German school system is divided into primary school (lasting four to six years, depending on the federal state) and secondary schools, which includes lower and higher track schools. Lower track schools generally do not lead to university entrance qualifications, while higher track schools typically do, although a switch from one system to the other is possible. Comprehensive schools offer both pathways (30). Special-needs schools exist separately. Compulsory schooling begins at age six, after which students are streamed into lower track or higher track secondary schools at age ten or twelve, depending on the federal state (30, 31). Some schools offer all-day programs (31). A persistent social gradient characterizes the system with lower track secondary and comprehensive schools enrolling a larger proportion of students from disadvantaged backgrounds, whereas higher track secondary schools comprise of students of higher socioeconomic status (31, 32). Moreover, resource allocation differs across school types, prompting concerns about how such inequities influence the provision of mental health support (33, 34).

Germany has a federal education system, which has led to regional variation in education policies, e.g. in regard to staffing including mental health staff and services (35). Furthermore, resources are distributed unequally, with wealthy federal states having more financial resources, while poorer federal states have fewer resources, affecting education spending (36, 37).

In light of current challenges for German schools, such as the rise in global crises, increasing numbers of students with refugee backgrounds, and widening socioeconomic disparities, calls for intensified mental health support in schools have been raised (38–40). One way that mental health is supported in schools is through professions specialized in mental health support. In German schools the most common mental health professionals are guidance counselors (41, 42), school psychologists (43, 44), and school social workers (45, 46). Guidance counselors are teachers with additional qualifications who are partially exempted from teaching duties to provide counseling (42). School psychologists hold psychology degrees, whereas school social workers are trained in social work (44, 47, 48) Both guidance counselors and school social workers conduct individual consultations, but with different professional backgrounds (42, 48). School social workers also work with groups and perform conflict resolution (48). School psychologists do counseling on more severe cases (44, 47, 49). While guidance counselors are part of the teaching body, school social workers, although stationed in schools, are employed outside the teaching staff (48, 49). School psychologists are typically hired by federal agencies and are responsible for multiple schools (44, 47). The hiring processes for guidance counselors, school psychologists, and school social workers vary across regions, involving federal, state, municipal, and school-level decision making authorities (45, 50, 51). Additionally, more targeted initiatives, such as school coaches and school nurses, complement these services (52, 53).

These resources are also unequally distributed between federal states and school types, although the extend of this inequality is still unclear (33, 34, 43, 54). There are significant research gaps in the field of mental health support in German schools. While statistics enumerate the number of school psychologists and school social workers employed by each German state, data on service demand remains absent (43, 54). Additional, one study examined differences by school type but only examined mental health services in aggregate rather than in terms of specific services, leaving many questions unanswered (33). The research is also complicated by the federal education system with its regional differences (43, 54).

### Limitations of previous research

Despite the critical role of schools in youth mental health, there is a lack of scientific research on schools as mental health stakeholders. There is some comparative research on school support structures, e.g., by Patalay and colleagues (27, 55) for European cross-national research, or Teich et al. (56) and Slade (57) for US research comparing states. Furthermore, there are numerous evaluations of specific programs [e.g., (58, 59)]. Such cross-national studies can identify particularly effective support structures and best-practice models.

Yet, systematic monitoring of school-based mental health services is lacking worldwide. Specifically, data on service provision remains unavailable, e.g. concrete data on services offered in an everyday school context, the extent of services (hours per week), and the demand for school-based support. Currently, demand for services can only be estimated via prevalence rates of mental health difficulties, but some affected youths also get help outside of schools, which makes it unclear who needs to be supported in schools [e.g., (11)]. Improved monitoring would enable evaluation of policy impacts and allow for data-driven adjustments [e.g., (60)].

At the national level, Switzerland offers one of the few examples of such monitoring (61). Zumbrunn et al. combined a literature review, staff interviews, and an online survey across 189 schools to assess service provision, needs, and challenges in respect to student mental health. Their findings reveal a demand for expanded mental health support in school and staff training. Existing services tended to be problem-specific, indicating a gap in general mental health prevention. Data on service demand as well as temporal changes and differences between school types is missing. Another relevant study was conducted in the U.S. in 2002–2003 and summarized by Teich and colleagues (56). They conducted a national survey of schools and school district that examined the prevalence and distribution of mental health services in public schools. The authors concluded that the frequency and severity of mental health problems were increasing while resource levels were static or decreasing. This study also lacks data on service demand, temporal changes, and differences between school types. Nevertheless, these studies provide a baseline for cross-national comparisons of school-based mental health. The present study aims to expand this corpus.

### The current study

To address the limited research on schools as mental health stakeholders, we conducted a survey study in Germany, which serves as the pilot phase of a larger long term longitudinal monitoring project, to examine the availability of and demand for mental health support systems in schools.

From a broader public health perspective, such a study may be of relevance, as it provides insights into the structural and contextual challenges of establishing mental health support systems in schools. Given that education and health governance differ markedly across countries, ranging from highly centralized to strongly federalized systems, Germany offers a valuable case study for understanding how institutional structures shape the provision and accessibility of school-based mental health services. The observed regional variation and lack of comprehensive demand-oriented data highlight issues that may also apply to other countries' school contexts, particularly those with decentralized education systems. Thus, these findings contribute to a comparative understanding of how schools can function as key stakeholders in mental health promotion, while underscoring the need for more standardized data collection across countries as a basis for service planning.

In the present study, we collected data on the provision of mental health services and their availability during the previous and current school years. In addition, we assessed the demand for such services and examined its development over the course of a school year, enabling a comparison between supply and demand. Furthermore, we analyzed variations across school types to identify potential disparities in access and provision.

Based on previous research, this study investigates the following hypotheses within the German school system:

Firstly, we expected that demand increased compared to the previous school year, operationalized through the corresponding questionnaire items (62, 63). Secondly, we hypothesized that the availability of mental health services remained unchanged compared to the previous school year (43, 64–67). This hypothesis was operationalized by (a) examining responses to the corresponding questionnaire item on guidance counselors and (b) comparing staffing numbers of school social workers between the 2022/23 and 2023/24 school years. Thirdly, we expected that the majority of schools have access to guidance counselors, school social workers, and school psychologists, operationalized through corresponding questionnaire items (42, 44, 45). Fourthly, we hypothesized that demand for mental health services exceeds provision. This hypothesis was operationalized by (a) calculating the discrepancy between provision and demand items for guidance counselors and school social workers and (b) examining the corresponding item for school psychologists (43, 68). Finally, we expected that higher track secondary schools offer greater access to mental health services than other school types, measured by calculating the differences in provision numbers between school types (33, 34).

## Materials and methods

### Study design and sample

Data was collected online using *Unipark*^®^ survey software from April to June 2024 in thirteen German federal states with permission from the federal education offices. The states of Bavaria, Saxony, and Saarland declined to be surveyed. The study was approved by the local ethics committee. No incentives were offered, but participants could elect to receive the study results. Schools were selected from a dataset from choicelab (69) containing all public schools in Germany (*N* = 24,865). A total of 8,998 schools were chosen at random and another 92 because of their participation in a government initiative which was part of a different research project. We sent a letter with details on the study, a URL, and a QR code to a total of 9,090 schools. Some letters could not be delivered (*n* = 206). Out of 8,884 successfully contacted schools, 739 participated in the study. The response rate was 8.3%. In the course of data analysis, 253 schools were excluded: 244 because they did not complete the questionnaire, 6 vocational schools and 3 that were not schools.

The sample size for analysis was *N* = 486 schools from 13 federal states (see Table 1). Due to low sample sizes, data from Berlin, Hamburg, and Bremen was aggregated to preserve anonymity. The majority were primary schools (“Grundschulen”; *n* = 249; 51.2%), followed by lower track secondary and comprehensive schools, that were merged for analysis (“Haupt-/Real-/Gesamtschulen”; *n* = 113; 23.3%). Special-needs schools (“Förderschulen”) constituted 14.0% of the sample (*n* = 68) and higher track secondary schools 11.5% (“Gymnasien”; *n* = 56).

**Table 1:** Survey participants by federal state.

Federal state	*n*	Percentage (%)
Brandenburg	29	6.0
Baden-Württemberg	118	24.3
City states (Berlin, Hamburg, Bremen)	22	4.5
Hesse	29	6.0
Lower Saxony	107	22.0
Mecklenburg-West Pomerania	11	2.3
North Rhine-Westphalia	66	13.6
Rhineland-Palatinate	35	7.2
Saxony-Anhalt	15	3.1
Schleswig-Holstein	25	5.1
Thuringia	29	6.0

Unweighted data.

Special-needs schools enrolled the fewest students on average (*M* = 162.6; *SD* = 104.2), followed by primary schools (*M* = 222.2; *SD* = 119.1), and lower track secondary and comprehensive schools (*M* = 591.0; *SD* = 358.6). Higher track secondary schools had the highest average enrolment (*M* = 757.0; *SD* = 249.7).

The schools were distributed across municipalities of varying population sizes: 14.1% (*n* = 69) were located in municipalities with less than 5.000 inhabitants, 12.7% (*n* = 62) in those with 5.000–9.999 people, 17.5% (*n* = 85) in 10.000–19.999, 22.6% (*n* = 110) in 20.000–49.999, 11.2% (*n* = 54) in 50.000–99.999, 13.7% (*n* = 67) in 100.000–499.999 and 8.1% (*n* = 39) in municipalities with 500.000 or more residents.

### Measures

The survey was the pilot phase of a national monitoring project on mental health support structures in schools. As part of this research, a survey was developed in a participatory process through a literature review and interviews with eleven experts and pilot testing by an interdisciplinary expert committee as there was no appropriate measure available for German school mental health services. We interviewed school administrators, school social workers, school psychologists, guidance counselors, and employees of federal school agencies. The survey has some methodological overlap with the study conducted by Zumbrunn et al. (61), but is tailored to the German school system. The online questionnaire included questions on school characteristics and different forms of mental health support, as well as other variables not relevant to the article. The survey was administered in German and consisted of 91 questions, taking about 30 min to complete. Questions were both multiple choice and open ended (see Supplementary material).

#### Sociodemographic information

The survey included questions on the school type, federal state, and number of students. Principals estimated the percentage of their students who had special education needs, were from families receiving government financial assistance, and spoke little or no German. For analysis of German school types, lower-track secondary and comprehensive schools were combined.

#### Mental health support

Schools were asked whether they employed guidance counselors or social workers (yes/no). In this study, guidance counselors were defined as teachers with additional qualifications who were partially exempted from teaching duties to provide counseling. If present, schools reported the number of counseling sessions offered per week, changes in capacity between the 2022/23 and 2023/24 school years, and estimated the number of sessions required to meet demand. Responses were recorded as whole sessions per week. Service provision was operationalized by the presence of guidance counselors and the number of sessions offered, whereas demand was measured by the number of sessions required.

For school social work, schools reported the number of full-time equivalent (FTE) school social work positions for the 2022/23 and 2023/24 school years, as well as an estimation of the FTEs required to meet demand. Schools also indicated the number of temporary positions, reported in FTEs. FTE was defined for respondents as a position with 40 working hours a week and answers were provided as fractional values. The provision of school social work services was operationalized by the presence of social workers and the number of FTE positions, whereas demand was measured by the number of FTEs required to meet it.

Regarding school psychology, schools were asked whether they had access to school psychology services (yes/no), which measured service provision. If so, they specified whether services were provided by externally employed psychologists or by psychologists employed by the school on a temporary or permanent basis. Schools were also asked whether they had an assigned psychologist at the federal bodies responsible for school psychology in a yes/no format. Their accessibility by phone was estimated on a 6-point Likert scale (1 = “very difficult” to 6 = “very easy”), with an additional option for “never used service before/do not know”.

Schools were asked to estimate changes in demand for all forms of mental health support since the previous school year using a 5-point Likert-scale (1 = “strong decrease” to 5 = “strong increase”), with an additional “do not know” option. They also rated whether existing services met demand on a 4-point Likert scale (1 = “no” to 4 = “yes”), measuring the difference between provision and demand for services. In cases where schools lacked guidance counselors, school social workers, or access to school psychology services, they were asked whether there was a demand for the respective type of support (yes/no). In total, the survey included seven questions on guidance counselors, nine on school social work, and 21 on school psychology.

### Hypothesis operationalization

The first hypothesis was assessed through the corresponding questionnaire items on how demand changed compared to the previous school year (items 6f, 7f and 8e; see Supplementary material). The second hypothesis was operationalized by (a) examining responses to the corresponding questionnaire item on how the amount of guidance counseling sessions changed compared to the previous school year (item 8c) and (b) comparing staffing numbers of school social workers between the 2022/23 and 2023/24 school years. The third hypothesis was measured by the corresponding questionnaire items on whether schools had access to guidance counselors, school social workers, and school psychologists (items 6, 7 and 8). The fourth hypothesis was operationalized by (a) calculating the discrepancy between the provision and demand items for guidance counselors and school social workers and (b) examining the corresponding item for school psychologists on whether demand was covered by existing service provision (item 6b). The fifth hypothesis was measured by calculating the differences in provision numbers between school types.

### Data analysis

Data was weighted through Iterative Proportional Fitting by school form and federal state using data from the Federal Statistical Office of Germany to ensure the representativeness of results (70). The distribution of school types across each state in the sample was aligned with the corresponding actual statewide distributions. The individual weights ranged from 0.34 to 2.16. A power analysis was conducted to confirm sufficient statistical power for detecting the anticipated effects. Power analysis for chi-square analysis showed a minimum sample size of *n* = 121 to detect a medium effect (*w* = 0.3) with a significance level of *p* < 0.05 and a power of *p* (1-β) = 0.8. For ANOVA, the group sizes had to be at least *n* = 31 and for *t*-test the number of pairs at least *n* = 89.

Complete case analysis was used because questionnaire dropouts rendered the data unusable under consent restrictions. All variables were analyzed descriptively after the number and demand for counseling sessions and school social worker positions were standardized to a denominator of 500 students to get per-capita information. School social worker positions were recorded as full-time equivalents (40 h per week). Extreme values were excluded for logical reasons if they were implausible (e.g. if there were more guidance counseling sessions than students).

Some analyses were performed in subsamples. The full sample was used to examine whether schools offered guidance counseling, social work, or psychology services and how demand for these services changed relative to the preceding school year. For each service, subsequent analyses were limited to the subsample of schools that reported providing that service: (a) number, demand, and changes in guidance counseling sessions; (b) number of school social worker positions in the current and past school years (temporary vs. permanent) and the corresponding demand; (c) variables pertaining to school-psychologist provision. In addition, subsamples which reported not offering one of a given service were analyzed for their demand for that service.

To assess the difference between supply and demand for guidance counseling sessions and school social worker positions, as well as differences in supply of school social worker positions between school years, *t*-tests were used. Furthermore, group differences by school type were investigated. Chi-square tests were used for categorical variables, with effect sizes calculated as Cramer's *V* (with 0.10 indicating a small, 0.30 a medium and 0.50 a strong effect). As *post-hoc* test, we performed chi-square analysis between individual groups with Bonferroni correction. For metric variables, analyses of variance (ANOVAs) were performed, with eta-squared as effect size and Tukey's honest significance test (Tukey's HSD) as a *post-hoc* test. Where sample sizes were insufficient for formal analysis, exploratory analyses were conducted and explicitly labeled as such.

The significance level for all analyses was set at *p* < 0.05. All analyses were performed using R version 4.3.0. Analyses were performed with weighted and unweighted data. Reported results other than sample characteristics are weighted according to sociodemographic characteristics of the German population.

## Results

### Sample description

Surveyed schools had an average of 361.5 students (*SD* = 302.5), closely mirroring the German average of 333 students (71–73). Principals estimated that 18.4% of their students had special education needs (*SD* = 32.6), exceeding the German national average of 6.8% (71, 74). After applying sample weights, the estimated prevalence declined to 13.6% (*SD* = 26.5). Schools reported that an average of 24.6% (*SD* = 19.4) of their students were from families receiving government financial assistance, aligning closely with official estimated that approximately one-quarter of children and adolescents receive such support (75). Principals estimated that 12.9% of their students spoke little or no German (*SD* = 15.1), which mirrors official statistics showing that ca. 15% of people in Germany speak little or no German at home (76). Special education needs, families receiving government financial assistance and students speaking little or no German by school type are depicted in Table 2.

**Table 2:** Sociodemographic information by school type.

Sample characteristic	Percentage (%)	*SD*
Primary school (*n* = 249)		
Students with special education needs	5.5	6.6
Families receiving government financial assistance	21.1	17.7
Students speaking little or no German	13.7	15.8
Lower track secondary and comprehensive school (*n* = 113)		
Students with special education needs	8.3	7.0
Families receiving government financial assistance	26.2	16.9
Students speaking little or no German	14.4	16.2
Higher track secondary school (*n* = 56)		
Students with special education needs	1.6	2.0
Families receiving government financial assistance	11.1	8.1
Students speaking little or no German	2.9	3.1
Special-needs school (*n =* 68)		
Students with special education needs	96.8	14.6
Families receiving government financial assistance	44.9	20.2
Students speaking little or no German	15.7	14.0

Unweighted data.

### Development of demand and availability of mental health support

Most surveyed schools reported that the demand for different forms of mental health support had increased “slightly” or “strongly” since the previous school year (66.5%−71.5%). All data in Table 3.

**Table 3:** Development of demand for different mental health services between the school years of 2022/23 and 2023/24.

Mental health service	Strong increase	Slight increase	No change	Slight decrease	Strong decrease	No comment
Guidance counseling sessions	26.6%	39.9%	27.3%	0.8%	0.4%	5.1%
School social worker positions	37.7%	33.8%	26.1%	1.0%	0.5%	0.9%
School psychology services	34.0%	34.3%	23.5%	0.5%	0.6%	7.1%

In terms of availability, most principals (57.4%; *n* = 106) reported the number of weekly guidance counseling sessions remained unchanged between the previous school year and the school year of the survey. A minority of 4.1% (*n* = 8) reported a “slight” and 1.3% (*n* = 2) a “strong” decrease, conversely 19.7% (*n* = 36) a “slight” and 11.6% (*n* = 21) a “strong” increase; 5.9% of responses (*n* = 11) were missing.

Similarly, the average number of school social worker positions did not differ significantly between school years (both *M* = 1.6; *p* = 0.458).

### Guidance counselors

Across all school types, 184 schools (37.9%) reported having guidance counselors on staff. Chi-square test [χ^2^(3) = 84.16; *V* = 0.42; *p* < 0.001] showed higher availability of guidance counselors in lower track secondary and comprehensive schools (60.7%; *n* = 73) and higher track secondary schools (74.1%; *n* = 37) than in primary schools (22.0%; *n* = 60) and special-needs schools (32.4%; *n* = 14).

Out of all schools without guidance counselors, 220 (72.8%) reported a demand for this form of mental health assistance. Demand differed between school types in exploratory analysis [χ^2^(3) = 12.10; *V* = 0.20; *p* = 0.007], with primary schools (75.1%; *n* = 160) and lower track secondary and comprehensive schools (81.0%; *n* = 38) reporting a higher demand than special-needs schools (48.4%; *n* = 14). Higher track secondary schools' demand did not differ from other school types (59.3%; *n* = 8).

The reported average weekly number of counseling sessions was 6.4 (*SD* = 12.4; 95% CI = 4.4–8.3) per 500 students across all school forms, which did not meet the reported weekly demand of *M* = 13.5 (*SD* = 23.6; 95% CI = 9.7–17.2) sessions per 500 students. The reported number of counseling sessions and demand differed significantly [*t*(272) = −3.6; *p* < 0.001; *r* = 0.84]; for details, see Table 4.

**Table 4:** Weekly counseling sessions: number and demand.

Counseling session	*M*	*SD*	95% CI
Primary school (*n* = 60)			
Counseling session number	8.7	11.0	5.6 to 11.9
Counseling session demand	19.6	29.3	11.2 to 28.1
Margin of number and demand	−10.9	20.4	−16.8 to −5.0
Lower track secondary and comprehensive school (*n* = 73)			
Counseling session number	3.5	4.1	2.5 to 4.6
Counseling session demand	8.6	11.3	5.6 to 11.6
Margin of number and demand	−4.9	8.9	−7.3 to −2.6
Higher track secondary school (*n* = 37)			
Counseling session number	3.1	2.5	2.2 to 3.9
Counseling session demand	6.1	3.3	5.0 to 7.2
Margin of number and demand	−3.5	2.7	−4.4 to −2.5
Special-needs school (*n =* 14)			
Counseling session number	22.2	37.1	2.3 to 42.1
Counseling session demand	32.5	49.3	6.8 to 58.2
Margin of number and demand	−13.3	24.7	−26.5 to 0

Weekly counseling sessions per 500 students.

The number of weekly counseling sessions differed between school types (*p* < 0.001; η^2^ = 0.156), with special-needs school reporting higher numbers than all other school types (*p* < 0.001). Additionally, lower track secondary and comprehensive schools reported lower numbers than primary schools (*p* = 0.0497). In exploratory analysis, the demand for counseling sessions also differed between school types (*p* < 0.001; η^2^ = 0.107). Special-needs schools (*p* < 0.001) and primary schools (*p* < 0.02) reported higher demand than lower track secondary and comprehensive schools and higher track secondary schools.

### School social work

Across all school types, 366 schools (75.3%) reported they had school social workers on staff. The availability of school social workers differed by school type [χ^2^(3) = 36.27; *V* = 0.27; *p* < 0.005]. Primary schools (70.5%; *n* = 192), higher track secondary schools (62.1%; *n* = 31), and special-needs schools (64.8%; *n* = 28) reported lower availability than lower track secondary and comprehensive schools (95.2%; *n* = 115). On average, 25.7% of full-time equivalent school social workers positions were temporary, whereas 74.3% were permanent. Some principals did not know whether positions were temporary or permanent (*n* = 51).

Out of all schools without school social workers on staff, 108 (89.6%) reported a demand for this service. Demand for school social workers did not differ between school forms in an exploratory chi-square analysis (*p* = 0.824).

The average number of full-time equivalent school social worker positions per 500 students across all school types was 1.6 (*SD* = 1.2; 95% CI = 1.4–1.7), whereas the demand was estimated at 2.9 positions (*SD* = 2.0; 95% CI = 2.7–3.1); for details, see Table 5. Number and demand differed significantly [*t*(582) = −10.9; *p* < 0.001; *r* = 0.78].

**Table 5:** School social worker positions: number and demand.

School social worker position	*M*	*SD*	95% CI
Primary schools (*n* = 192)			
School social worker positions number	1.5	1.0	1.4 to 1.7
School social worker positions demand	3.0	1.6	2.7 to 3.3
Margin of number and demand	−1.5	1.2	−1.7 to −1.3
Lower track secondary and comprehensive school (*n* = 115)			
School social worker positions number	1.6	1.2	1.4 to 1.9
School social worker positions demand	2.7	2.1	2.3 to 3.2
Margin of number and demand	−1.1	1.3	−1.4 to −0.9
Higher track secondary school (*n* = 31)			
School social worker positions number	0.7	0.4	0.5 to 0.8
School social worker positions demand	1.1	0.5	0.9 to 1.2
Margin of number and demand	−0.4	0.3	−0.5 to −0.3
Special-needs school (*n =* 28)			
School social worker positions number	2.7	2.4	1.9 to 3.5
School social worker positions demand	4.9	3.5	3.8 to 6.0
Margin of number and demand	−2.3	2.0	−2.9 to −1.6

Full-time equivalent positions per 500 students.

The number of (*p* < 0.001; η^2^ = 0.111) and demand for school social worker positions (*p* < 0.001; η^2^ = 0.147) differed between school types, with all school types differing from each other (*p* ≤ 0.001) except primary schools and lower track secondary and comprehensive schools.

### School psychology

Across all school types, 371 schools (76.4%) had access to services by school psychologists. The availability did not differ between school types (*p* = 0.653). Out of all primary schools, 74.5% (*n* = 203) reported they had access to school psychology services, out of lower track secondary and comprehensive schools 79.2% (*n* = 96), higher track secondary schools 75.7% (*n* = 38), and special-needs schools 81.0% (*n* = 35).

Only nine principals (2.4%) stated they had a school psychologist on staff in their school. Furthermore, 54.8% of schools (*n* = 198) had a psychologist appointed by the government agency that oversees school-psychology services. The rest did not have an assigned staff member (30.1%; *n* = 109), did not know whether they had an assigned staff member (6.2%; *n* = 23), or did not comment (8.9%; *n* = 32). There was no difference between school types (*p* =.132).

Out of all schools without access to school psychology services, 109 (94.7%) reported a demand for these services. Demand for school psychology services did not differ between school forms in an exploratory chi-square analysis (*p* = 0.900).

Most principals (41.9%; *n* = 156) stated demand for school psychology services was not met by existing services, followed by “not really” (35.0%; *n* = 130), “somewhat” (18.3%; *n* = 68), and “yes” (4.8%; *n* = 18). There was no difference between school types in exploratory analysis (*p* = 0.539).

When principals were asked how easy it was for them to reach the federal bodies for school psychology on a case-by-case basis via telephone, the most common response was that they were “somewhat easy” to reach (27.8%). All data is presented in Table 6.

**Table 6:** Assessment of accessibility of the federal bodies for school psychology via telephone.

How difficult is it to reach the federal body responsible for school psychology via telephone?	Percentage (%)	*n*
Very difficult	4.0	14
Mostly difficult	6.5	24
Somewhat difficult	22.0	80
Somewhat easy	27.8	101
Mostly easy	21.2	77
Very easy	4.5	16
Never used service before/do not know	5.1	18
No comment	8.9	32

## Discussion

Given the high prevalence of mental disorders among children and adolescents, schools are increasingly recognized as critical settings for prevention and intervention (11, 15). Despite this growing recognition, evidence suggests that mental health support structures in most European schools are inadequate, though comprehensive data remains limited (77). To address this gap, we surveyed 486 German schools on their mental health support infrastructure in the 2022/23 and 2023/24 school years. We hypothesized that demand would increase compared to the previous school year while service provision would remain unchanged, that the majority of schools would have access to guidance counselors, school social workers, and school psychologists, and that demand would exceed existing service provision. Finally, we expected higher track secondary schools to offer greater access to mental health services than other school types. Results indicated that demand across all service types increased compared to the previous school year, whereas service provision remained unchanged. While most schools reported having school social workers and (remote) access to school psychology services, fewer than half employed guidance counselors. Where services were absent, demand was consistently high. Overall, service provision failed to meet demand, which was up to twice as high as that offered by the services available. While resource accessibility varied by school type, no consistent advantage was observed across school categories, though special-needs schools reported the highest levels of both service provision and demand.

### Development of demand and availability of mental health support

Our first hypothesis, that demand would increase relative to the previous school year, was confirmed. Reported demand for all types of mental health services increased, likely reflecting elevated prevalence rates of mental health problems and the impact of ongoing global crises (11, 78, 79). Given the limited capacity of external mental health providers, as evidenced by prolonged waiting times for outpatient psychotherapy placements, students may instead be increasingly seeking support within schools (80).

Our second hypothesis, that the provision of mental health services would remain unchanged relative to the previous school year, was supported for both guidance counseling hours and school social worker positions, which showed no significant change relative to the previous school year. The persistence of an unchanged level of mental health service provision concurrent with a rising demand is troubling, as it signals an expanding disparity between service availability and student needs, thereby exacerbating the adverse conditions experienced by the student population.

### Service provision

Our third hypothesis, that the majority of schools would have access to guidance counselors, school social workers, and school psychology services, was only partially confirmed. Most schools reported employing school social workers, with an average of one and a half full time positions. However, a quarter of school social worker positions were reported as temporary, suggesting positions are precarious and contingent on the school's financial situation and political staffing policies. The majority of schools also reported access to school psychology services, although provision was predominantly remote and direct employment of psychologists at schools was rare, with less than three percent of schools reporting having an internal school psychologist. Half of the schools reported being aware of an assigned psychologist within the relevant state body. The accessibility of external school psychologists via telephone varied, with most respondents characterizing contact as “somewhat difficult”, “somewhat easy”, or “mostly easy”.

In contrast, fewer than half of the schools reported employing guidance counselors, pointing to a concerning gap in service provision. The findings may have also resulted from the study's narrow definition of guidance counselors, which required formal counseling qualifications. In practice, counseling tasks are often performed by teachers without specialized training, suggesting that a broader definition may have yielded higher reported availability (49, 81, 82). Further research should differentiate between trained and untrained guidance counseling positions. Where guidance counseling was present, schools delivered an average of six and a half sessions per week.

When compared to earlier national research, service availability seems to have increased. In 2002, Mack (33) found that fewer than half of German schools reported offering any mental health services, although the study did not differentiate among service types.

The quality of mental health support in schools depends not only on staff quantity but also on staff qualifications, which critically affect service effectiveness. Consequently, future research should incorporate indicators of personnel training and service quality.

### International comparisons

Cross-national comparisons must be interpreted cautiously because of differing definitions, measurements, and data sources. In a Swiss study by Zumbrunn et al. (61), 67.7% of Swiss schools employed school social workers, which is slightly below the German figure of 75.3%. Conversely, 83.0% of Swiss schools reported “often” or “rare” contact with school psychologists, compared to 76.4% in our study, suggesting different national priorities in service provision. Compared with the U.S. study by Teich et al. (56), German schools employed markedly fewer school counselors (37.9 vs. 77% in the U.S.). However, German schools had more contact with school psychologists (76.4 vs. 68% in the U.S.) and employed more school social workers (75.3 vs. 44% in the U.S.). Notably, the U.S. sample also included a distinct professional group, with 69% of schools employing school nurses.

School nurses predominate also in several Nordic and Central-European systems. Van der Pol (2020), employing a mixed-method approach of expert surveys and publicly available statistics, reported >1 nurse per 1,000 students in Norway, Austria, and Finland, whereas psychologist ratios were 0.1, 0.1, and 0.3 per 1,000 students, respectively. Norway also exhibited < 0.01 social workers per 1,000 students (83).

The International Network for School Social Work's cross-national expert survey documented a wide European range in school social worker staffing, from a total number of five workers in Liechtenstein to 2,991 in France^1^. Even among countries with comparable student populations, allocations differ markedly (e.g. Hungary ≈ 50 vs. Switzerland ≈ 900 social workers), reflecting distinct policy priorities. However, the lack of recent and school-level data hampers comparison.

The extent of the shortage of school psychologists in Germany becomes particularly evident when examined against European standards. Official statistics indicate that, on average, one school psychologist in Germany is responsible for 5,218 students (43). This number is considerably higher than in many other European countries. For example, in Finland, each psychologist attends to approximately 800 students (25). Earlier comparative data compiled by Jimerson et al. using a mixed-method synthesis of literature search, expert surveys, and public data likewise suggests that Germany occupies an unfavorable position in the European landscape, with student-to-psychologist ratios ranging from 773 in Denmark to 5,182 in Hungary (84). However, as these figures are more than a decade old, they offer only a limited basis for contemporary benchmarking. Reliable and up-to-date statistics are therefore needed to situate the German case within a broader international framework of mental health provision in schools.

Comparable limitations apply to data on guidance counselors. Across twelve European countries, Popov and Spasenović (85) estimated, via a document study, caseloads between 250 and 500 students per counselor, with Croatia exhibiting the most favorable ratio. More recent national data illustrates the scale of provision, such as the 3,120 public-school counselors reported in Swedish official statistics in 2023 (86). Yet, cross-national comparisons remain complicated by variations in role definitions, professional training, and institutional embedding (85). These divergences underscore the importance of assessing school-based mental health support systems as a whole rather than focusing exclusively on individual professional groups. Furthermore, variations in staff qualifications may affect service quality, a factor obscured when analyses consider only service provision counts. Nevertheless, the paucity of systematic and comparable data continues to impede international benchmarking.

### Service demand

Consistent with our fourth hypothesis, the reported demand for mental health services exceeded existing provision. This is shown by data on guidance counselor sessions and school social worker positions, which shows demand up to twice as high as existing service provision. Furthermore, accounts of school psychology services underscore these findings, with most principals stating demand was “not” or “not really” met by existing services. Schools lacking access to guidance counselors, school social workers, or school psychology services generally expressed high demand, with over three-quarters of the schools indicating a need for these services. This suggests that limited service provision was not due to a lack of need but was likely due to structural constraints. However, the precise mechanisms remain unidentified and the indicators were estimated, raising concerns about subjectivity and necessitating cautious interpretation.

These findings corroborate those reported by Mack (33), who surveyed adolescents aged 14–16 in three German municipalities and identified a comparable discrepancy between demand for and provision of services. The Swiss investigation by Zumbrunn et al. (61), which employed a methodological framework analogous to the present study, examined slightly different outcome variables. They classified services as “internal” or “external”, whereas our investigation employed a profession-based taxonomy. For internal services, Zumbrunn et al. reported merely on their presence within schools, for external services, they estimated contact frequency with outside agencies, while our study quantified guidance counseling sessions and school worker positions. Zumbrunn et al. also estimated demand for different services, but our study provided a more detailed account and assessed the extent to which that demand was satisfied. Nonetheless, the study by Zumbrunn et al. also revealed that schools expressed a heightened need for counseling and support not only for affected students but also for teachers and parents. In their conclusion, the authors recommend expanding services–conclusions that align with our findings. Moreover, a European Commission report, based on expert-survey analyses, indicated certain or severe personnel shortages in school health services across 30 European nations, including Germany, and recommended service expansion (77).

### Differences between school types

Our fifth hypothesis, which posited that higher track secondary schools would provide greater access to mental health services than other school types, was not supported. While these schools reported the highest availability of guidance counselors, this advantage reached statistical significance only in comparison with primary and special-needs schools. Conversely, higher track secondary schools exhibited the lowest availability of school social workers, the fewest counseling sessions and school social worker positions, and the lowest demand for these services, although not all differences were statistically significant. Access to school psychology services did not vary across school types. These findings partially contradict initial assumptions.

A critical contextual factor lies in the comparatively large enrolments of higher track secondary schools. When resources are standardized to a denominator of 500 students, these schools display the lowest per-capita provision of and demand for mental health services, even though their absolute resources and demand may exceed those of smaller institutions. This finding underscores the importance of accounting for student numbers in assessing service accessibility, as absolute staffing levels can obscure inequities in support provision.

Interestingly, special-needs schools reported both the highest provision and highest demand for guidance counseling sessions and school social worker positions, which could not be fully met through existing services. Elevated demand in this context is consistent with the heightened mental health needs of their student populations (87). However, their smaller enrolments inflate per-500-student metrics, potentially overstating the absolute numbers of both provision and demand.

Historical data further illustrates complex patterns. In 2002, “Realschulen” offered the highest rates of mental health services, followed by higher track secondary schools (33). In the present study, “Realschulen” were subsumed under the broader category of lower track secondary and comprehensive schools, limiting direct comparison and highlighting the heterogeneity within this group. Future research should therefore employ more granular classifications of secondary-school subtypes to disentangle intra-sectoral disparities.

Taken together, these findings highlight that the distribution of mental health services in German schools is shaped less by school type *per se* than by enrolment size, contextual needs, and historical developments. This pattern resonates with broader debates on equitable resource allocation, where the OECD (Organization for Economic Co-operation and Development) and WHO emphasize the principle of proportionality: services should be allocated according to student numbers and need profiles rather than institutional status alone (88). From a policy perspective, aligning German provision with these international standards would not only improve equity in access but also contribute to the realization of global commitments such as the UN Sustainable Development Goals, particularly SDG 3 (“Good Health and Wellbeing”) and SDG 4 (“Quality Education”) (89). However, the observed lower per-capita provision in higher track secondary schools constitutes a systematic misalignment with the OECD and WHO proportionality recommendation. By reorienting funding mechanisms toward empirically measured need, German education authorities can move toward a more equitable mental health service landscape.

### Limitations and future directions

Our study has several strengths. The data is representative, having been obtained through near-nationwide random sampling and weighted to enhance generalizability. Furthermore, to our knowledge, this is the first systematic collection of concrete service metrics (e.g. demand estimates, weekly counseling sessions) and thus provides a baseline for ongoing national monitoring. Students with special education needs were overrepresented, thereby mitigating the paucity of research on this cohort. Moreover, principals provided estimated, not clinically diagnosed, prevalence rates of special education needs, which may have led to a more accurate rate by including undiagnosed students.

However, several limitations must be acknowledged. Three federal states were omitted because state approval was not granted, reducing full-nation coverage. The overall participation rate was relatively low (8.3%), which constrains the external validity of the findings. The non-participation of the three federal states and low response rates are a potential source of non-response bias. Because the random sample did not include data on non-participating schools, a dropout analysis could not be conducted. Thus, potential systematic differences between participating and non-participating schools cannot be ruled out.

Additionally, the federal structure of the German school system complicates data collection, as service terminology and structure may differ from those used in the survey. Moreover, demand for mental health services was inferred rather than directly observed. The subjective nature of these estimates raises concerns about accuracy and respondent bias. There were no qualitative items on the variables reported, allowing divergent participant interpretations and hindering elaboration of responses. Moreover, some analyses were exploratory as sample sizes were insufficient for formal analysis. As most of these analyses pertain to special-needs schools, which are severely under-researched, these exploratory results remain valuable for highlighting differences across school types. Finally, differences between federal states were not examined because the sample size was insufficient. Given the substantial variation in financial and educational resources across federal states, this may have affected the results (36, 37).

The study has both practical and research implications. The study findings indicate a lack of demand-oriented planning. Mental health support structures should be expanded, particularly for schools with high demand, such as special-needs schools. This expansion should be accompanied by systematic monitoring in order to quickly assess results and adjust. Given the federalized nature of the German education system, coordinated intra-national exchange is essential.

This study is the first step toward international monitoring, with subsequent waves planned to track developments. It uniquely provides insight into the structural and contextual challenges of establishing mental health support systems in schools. Future waves should aim for larger sample sizes and assess resource disparities across German federal states. Furthermore, survey data should be supplemented with official statistics, where feasible. If more countries followed suit, we would be able to identify best-practice examples, solve the high demand-provision gap through mutual exchange and cement mental health support structure management as a core component of school planning. In conclusion, school mental health service provision remained unchanged relative to the previous school year despite rising demand. While most German schools reported access to mental health services through school social workers and external psychology services, service provision fell short of demand. Less than half of schools had access to a guidance counselor. Although the distribution of services varied across school types, none of the types demonstrated a systematic advantage. Consequently, mental health service provision in Germany should be expanded and an international monitor for school mental health services implemented.

## Data Availability

The datasets presented in this article are available from the authors upon reasonable request and with the permission of the Robert Bosch Foundation. Requests to access the datasets should be directed to Hannah Elisabeth Köhler, h.koehler@uni-leipzig.de.

## Footnotes

1 Huxtable M. International survey of school social work. International Network for School Social Work (2016). Unpublished Manuscript.
