# Supplementary material for: Mental health support in German schools: a cross-sectional survey study of principals on demand vs. provision of support services
Source: Front Public Health. 2026 Jun 24;14:1760608. doi: 10.3389/fpubh.2026.1760608 (PMC13341547; doi:10.3389/fpubh.2026.1760608)
Supplement: Supplementary file 1 [file Supplementary_file_1.docx]

**Supplementary Material**

**Questionnaire**

Only questions relevant to the article “Mental health support in German schools: a cross-sectional survey study of principals on demand vs. provision of support services” have been included.

1. School characteristics
2. What type of school is your school?

• Primary school

• Lower track secondary school, middle school, intermediate school, technical secondary school

• Secondary modern school

• Higher track secondary school

• Integrated comprehensive school, community school, integrated secondary school, district school

• School with multiple educational programs: cooperative comprehensive school, grammar school, secondary school plus, extended secondary school, regular school, regional school, secondary school

• Special educational school type: e.g. special needs school, special educational and counselling center

• Vocational school

• Other school type:

• It is not a school.

1. How many students are there at your school?

Please enter the number as an integer.

NUMERICAL ANSWER

1. What percentage of students at your school have recognized special educational needs?

NUMERICAL ANSWER

1. What percentage of students at your school come from families who receive government financial assistance in the form of unconditional basic income or housing allowance? Please estimate.

NUMERICAL ANSWER

1. What percentage of students at your school speak little or no German?

NUMERICAL ANSWER

1. Psychosocial support structures

*School psychology services*

1. Does your school offer school psychology services or could your school make use of such services?

Either through internal or external school psychologists.

- No
- Yes

IF “NO” ON QUESTION 6

1. Is there a demand for school psychology services at your school?

- No
- Yes

IF “YES” ON QUESTION 6

1. Do the school psychology services currently meet the demand at your school?

- No
- Not really
- Somewhat
- Yes

IF “YES” ON QUESTION 6

1. Who provides the school psychology services?

Please only refer to services provided by school psychologists.

*Note: The federal bodies responsible for school psychology are in charge of providing school psychology services in schools and may be referred to by different names in different federal states. Please refer to the federal body responsible for school psychology available in your federal state, e.g. school psychology service, central department for diagnostics and school psychology.

(multiple answers possible)

- External school psychologists from a federal body responsible for school psychology
- School psychologists working internally on a permanent basis at the school
- School psychologists working internally on a temporary basis at the school
- Other:

**Note:** The following questions relate to the federal body responsible for school psychology in charge of providing school psychology support in schools. This body has different names in each federal state. When answering the questions, please refer to the body that exists in your federal state (e.g. school psychology service, school psychology counselling centers, central department for diagnostics and school psychology, etc.).

IF “EXTERNAL SCHOOL PSYCHOLOGIST FROM A FEDERAL BODY RESPONSIBLE FOR SCHOOL PSYCHOLOGY” ON QUESTION 6 C)

1. How difficult is it to reach the federal body responsible for school psychology via telephone **currently**?

- Very difficult
- Mostly difficult
- Somewhat difficult
- Somewhat easy
- Mostly easy
- Very easy
- Never used service before/do not know

IF “EXTERNAL SCHOOL PSYCHOLOGIST FROM A FEDERAL BODY RESPONSIBLE FOR SCHOOL PSYCHOLOGY” ON QUESTION 6 C)

1. Is there a school psychologist assigned to your school at the federal body responsible for school psychology?

- No
- Yes
- Do not know

IF “YES” ON QUESTION 6 OR “NO” ON QUESTION 6 AND “YES” ON QUESTION 6 A)

1. How has the demand for school psychology services at your school changed this school year **compared to the previous school year**?

- Strong decrease
- Slight decrease
- No change
- Slight increase
- Strong increase
- Do not know

*School social work*

1. Are there school social work positions at your school?

- No
- Yes

IF “NO” ON QUESTION 7

1. Is there a demand for school social work positions at your school?

- No
- Yes

IF “YES” ON QUESTION 7

1. How many school social workers are there at your school **in the current school year**?

Please indicate the full-time equivalents in decimal numbers. If you do not know the exact number, please estimate. For example:

1.0 = One person working 40 hours per week or two people working 20 hours per week each

0.75 = One person working 20 hours per week and another person working 10 hours per week

1.5 = One person working 40 hours per week and another person working 20 hours per week

NUMERICAL ANSWER

IF “YES” ON QUESTION 7

1. How many school social workers at your school are employed on a fixed-term contract?

Please indicate the full-time equivalents in decimal numbers. If you do not know the exact number, please estimate.

- NUMERICAL ANSWER
- Do not know

IF “YES” ON QUESTION 7

1. How many school social workers were there at your school **in the previous school year**?

Please indicate the full-time equivalents in decimal numbers. If you do not know the exact number, please estimate.

- NUMERICAL ANSWER
- Do not know

IF “YES” ON QUESTION 7 OR “NO” ON QUESTION 7 AND “YES” ON QUESTION 7 A)

1. How many school social workers does/would your school **currently** need to meet demand?

Please indicate the full-time equivalents in decimal numbers. If you do not know the exact number, please estimate. For example:

1.0 = One person working 40 hours per week or two people working 20 hours per week each

0.75 = One person working 20 hours per week and another person working 10 hours per week

1.5 = One person working 40 hours per week and another person working 20 hours per week

NUMERICAL ANSWER

IF “YES” ON QUESTION 7 OR “NO” ON QUESTION 7 AND “YES” ON QUESTION 7 A)

1. How has the demand for school social workers at your school changed this school year **compared to the previous school year**?

- Strong decrease
- Slight decrease
- No change
- Slight increase
- Strong increase
- Do not know

*Guidance counsellors*

1. Are there guidance counsellors at your school? (Teachers with appropriate additional qualifications and partial exemption from teaching duties for counselling activities)

- No
- Yes

IF “NO” ON QUESTION 8

1. Is there a demand for guidance counsellors at your school?

- No
- Yes

IF “YES” ON QUESTION 8

1. How many weekly counselling sessions are **currently** available to the guidance counsellors at your schools for counselling activities?

If there are several positions, please add up the weekly counselling sessions.

Please enter the number as an integer. If you do not know the exact number, please estimate.

NUMERICAL ANSWER

IF “YES” ON QUESTION 8

1. How have the weekly counselling sessions of your school’s counselling teachers changed this school year **compared to the previous school year**?

- Strong decrease
- Slight decrease
- No change
- Slight increase
- Strong increase
- Do not know

IF “YES” ON QUESTION 8 OR “NO” ON QUESTION 8 AND “YES” ON QUESTION 8 A)

1. How many weekly counselling sessions from counselling teachers does/would your school **currently** need to meet demand?

Please estimate and enter the number as an integer.

NUMERICAL ANSWER

IF “YES” ON QUESTION 8 OR “NO” ON QUESTION 8 AND “YES” ON QUESTION 8 A)

1. How has the demand for guidance counsellors changed this school year **compared to the previous school year**?

- Strong decrease
- Slight decrease
- No change
- Slight increase
- Strong increase
- Do not know
